# Comparative Functional and Isokinetic Analysis between Implants with Posterior Stabilization and Rotating Hinge Total Knee Arthroplasty

**DOI:** 10.1055/s-0044-1779685

**Published:** 2024-03-21

**Authors:** Sandra Tie Nishibe Minamoto, Alan de Paula Mozella, Victor Rodrigues Amaral Cossich, Ubiratã Faleiro Gavilão, Heitor Schuabb Machado, João Maurício Barretto

**Affiliations:** 1 Centro de Cirurgia do Joelho do Instituto Nacional de Traumatologia e Ortopedia. Rio de Janeiro, RJ, Brasil; 2 Grupo de Joelho do Hospital São Vicente, Rede D'Or, Rio de Janeiro, RJ, Brasil

**Keywords:** arthroplasty, replacement, knee, osteoarthritis, knee, muscle strength

## Abstract

**Objective** : To compare the function and muscle strength of the limb between patients undergoing knee arthroplasties using primary implants with posterior stabilization (control group) and patients with rotating hinge implants (Hinge group).

**Methods** : Function assessment was performed using the Knee Society Score (KSS) and muscle strength using an isokinetic dynamometer using a speed of 60°/s.

**Results** : 43 patients were analyzed, who underwent 51 surgeries, with the Hinge group comprising 25 surgeries and the control group comprising 26 primary surgeries. We did not observe significant differences between the Hinge and control groups in the values of functional KSS (p = 0.54), objective KSS (p = 0.91), peak flexor torque (p = 0.25) and peak extensor torque (p = 0.08). Patients in the Hinge group who underwent primary arthroplasties had a higher peak flexor torque (0.76 Nm/kg) than those who used the implant in revision after septic failure (0.33 Nm/kg) (p < 0.05). The constrained implant was indicated in arthroplasty revision surgeries with severe ligament instability and in cases of complex primary arthroplasties with bone destruction or severe coronal deformity in the coronal plane.

**Conclusion** : The use of constrained implants enables joint function and muscle strength comparable to patients who underwent primary arthroplasty using conventional implants with posterior stabilization. Patients undergoing septic revision with a rotating Hinge prosthesis exhibit lower flexor muscle strength compared to those undergoing primary arthroplasty with a constrained implant.

## Introduction

Total knee arthroplasty (TKA) is a surgical procedure for replacing the knee joint with metal prostheses. It is often indicated for the treatment of severe cases of osteoarthritis, providing good or excellent results in more than 90% of patients. Normally, for primary surgeries, implants with less constriction are used, which can retain or replace the posterior cruciate ligament. In cases of complex primary surgeries or TKA revisions, implants with a greater degree of constriction are more frequently used, which may be semi-constricted or constricted (Hinge type implants). 1 Currently, severe ligament instability, massive bone destruction, ligament hyperlaxity, severe fixed deformity in the coronal plane, severe arthrofibrosis, septic arthroplasty revision, severe rheumatoid arthritis and comminuted fracture in osteoporotic bone may be an indication for the use of Hinge implants. 2 3

These implants were designed by Walldius, 4 in 1953, for reconstruction after tumor resection. 4 5 However, the first generation prostheses evolved with limited clinical results, a high number of failures, limited durability and significant bone loss for revision. 6 In the second generation, despite the improvements observed in the design, the clinical results remained limited and, still, a high number of complications were observed. 7 Currently, we have the third generation of these implants, with changes in the design and use of rotating polyethylene components aiming for better distribution load and joint kinematics, as well as reducing stress at the implant-host bone interface. 8 Therefore, it is believed that modern constrained implants enable better clinical results; however, a limited number of studies evaluate the functional results of these implants, especially in the Brazilian population, and we also observed a reduced number of studies comparing the results of implants with different degrees of constriction. 9 10

Therefore, the objective of the present study was to compare the function and muscle strength of the limb between patients undergoing surgery using primary implants with posterior stabilization and patients with rotating hinged implants. We believe that patients with Hinge implants will have lower limb muscle strength and/or worse joint function, potentially related to the implant, but also due to the greater complexity of primary cases or because they are used in patients undergoing revision TKA, therefore, with several previous surgeries.

## Materials and Methods

From December 2009 to May 2016, 67 rotating constrained implants from the Rotation Hinge Knee (RHK) Zimmer-Biomet® system were used in 65 patients at our institution. Of these, 18 cases were complex primary arthroplasties, and 50 were total knee arthroplasty (TKA) revisions. For the present study, 24 patients who underwent 25 surgeries with Hinge-type implants were analyzed, constituting the "Hinge group". Inclusion criteria were patients undergoing TKA at the institution requiring Hinge-type constrained implants and having isokinetic evaluation. Exclusion criteria were: patients with less than one year of postoperative follow-up during the study period, non-compliance with postoperative guidelines, loss to follow-up, unfavorable outcomes with implant loss (amputation, arthrodesis), death for any reason, presence of muscle injuries, neurological injuries, extensor mechanism injuries and/or fractures in lower limbs, uncompensated systemic clinical disease, and patients residing outside the state where the study was conducted. 41 patients were excluded from the study, and the reasons were: loss to follow-up (15 patients), residence outside the state (8 patients), death (6 patients), lack of clinical conditions for isokinetic evaluation (5 patients), septic arthroplasty failure (3 patients), lack of interest in participating in the research (2 patients), and extensor mechanism failure (2 patients).

Out of the 25 surgeries in the Hinge group, 9 were primary arthroplasties, and 16 were TKA revisions. In 4 cases of primary arthroplasties, constrained implants were necessary due to bone destruction, and in 5 other cases, due to severe deformity in the coronal plane. Out of the 16 revisions, 11 were due to mechanical failures, and 5 were due to infection. The control group consisted of patients undergoing TKA with conventional primary implants with posterior stabilization, matched for age, sex, and body mass index (BMI) to the study group that used the constrained Hinge prosthesis.

Therefore, this is a retrospective case series conducted with 24 patients undergoing 25 TKAs using constrained implants with a rotating tibial platform and 19 individuals undergoing primary arthroplasties in 26 surgeries using implants with posterior stabilization. The study was previously approved by the Research Ethics Committee of the institution (CAAE: 47473315.4.0000.5273), and there was no age limit or restriction on the participants' gender.

In both groups, the following were analyzed: age, sex, follow-up time, weight, height, and BMI. The Hinge group was divided into the following subgroups: patients undergoing primary arthroplasty (primary group), patients undergoing revision after failure due to infection (septic revision group), and patients undergoing revision after aseptic failure (aseptic revision group).

The following analyses were performed between the control group and the Hinge group and between the subgroups of the Hinge group. Function evaluation was conducted through the application of the Knee Society Score (KSS), validated for the Portuguese language, consisting of an objective and a functional part. 11 The objective KSS analyzes pain, range of motion, joint stability, presence of stiffness, extension deficit, and alignment of the limb in the coronal plane. The functional part assesses the patient's mobility, the ability to go up and down stairs and the need to use aids for walking. The questionnaire was administered by two orthopedists during the postoperative follow-up consultation.

Muscle strength was analyzed using an isokinetic dynamometer (CSMI, model HUMAC NORM). To determine the maximum isokinetic voluntary strength, a concentric-concentric isokinetic test was performed for knee flexion and extension. The speed used was 60°/s and five repetitions were performed. The highest instantaneous torque found was considered the peak torque (PT) and used for the analyses. PT was standardized by body weight to enable better comparison between individuals.

## Statistical analysis

Descriptive analyses for quantitative data were conducted, presenting means, standard deviations (SD), medians, minimum and maximum values. The Shapiro-Wilk normality test and Levene's homogeneity test were employed. To compare the control and Hinge groups, the independent samples t-test was used, and when necessary, the non-parametric Mann-Whitney test. To analyze variables among the subgroups of the Hinge prosthesis, Analysis of Variance (ANOVA) and the non-parametric Kruskal-Wallis test were employed. When multiple mean comparisons were needed, the Bonferroni post hoc test and Dunn's post hoc test were used. Categorical variables were analyzed using the Chi-Square test or Fisher's Exact test when necessary. All analyses were conducted using SPSS 21 software for Windows with a significance level of α = 0.05.

## Results

The demographic data of the Hinge and control groups are described in Table 1 and the groups were similar in terms of age, weight, height, BMI, gender distribution and length of follow-up.

**Table 1:** Demographic data and follow-up time of patients undergoing knee arthroplasty with Hinge-type implant and implant with posterior stabilization (control group)

Variable	Hinge (n = 25)	Control Group (n = 26)	p value
**Age (years)**	68,20 ± 9,09	66,40 ± 6,19	0,23
**Sex**			
** Male**	4	5	0,76
** Female**	21	21	
**Wight (kg)**	78,42 ± 16,42	78,90 ± 12,95	0,66
**Height (m)**	1,59 ± 0,10	1,63 ± 0,09	0,27
** BMI (kg/m ^2^ ) **	31,19 ± 6,46	29,51 ± 3,97	0,61
**Follow-up time (months)**	32,74 ± 21,23	27,91 ± 18,41	0,37

There was no statistically significant difference regarding distribution by sex, age and length of follow-up between patients undergoing Hinge in primary surgery or revision surgery. The mean BMI presented different values between the groups (p = 0.02) ( Table 2 ) and, after performing the Bonferroni post hoc test, a p value < 0.05 was observed only between the septic revision and aseptic revision; therefore, the septic revision subgroup (38.41 kg/m2) had a higher average than the aseptic revision subgroup (28.37 kg/m2).

**Table 2:** Demographic data and follow-up time of patients undergoing knee arthroplasty with Hinge-type implant and implant with posterior stabilization (Control Group)

Variable	Primary (n = 9)	Sceptic Revision (n = 5)	Aseptic Revision (n = 11)	p value
**Age (years)**	66,73 ± 10,47	71,53 ± 7,02	67,81 ± 9,26	0,23
**Sex**				
** Male**	3	0	1	0,18
** Female**	6	5	10	
**Wight (kg)**	75,54 ± 15,46	90,52 ± 22,09	75,27 ± 19,78	0,29
**Height (m)**	1,57 ± 0,11	1,53 ± 0,10	1,62 ± 0,09	0,26
** BMI (kg/m ^2^ ) **	30,63 ± 6,73	38,41 ± 7,02	28,37 ± 5,43	0,02*
**Follow-up time (months)**	29,33 ± 25,22	35,51 ± 23,39	34,28 ± 18,33	0,62

**Fig. 1:**
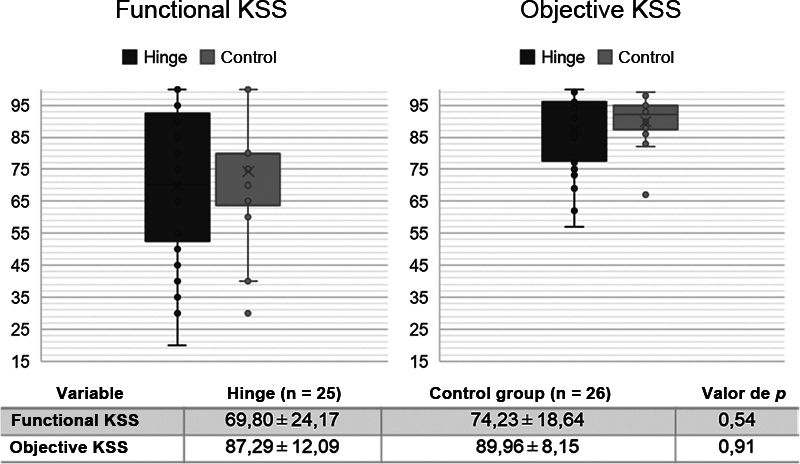
Functional and objective clinical scores (KSS) of patients undergoing total knee arthroplasty with Hinge-type prosthesis (Hinge: dark gray) and with prosthesis with posterior stabilization (Control: light gray). KSS represented in dots. SD = Standard deviation. Mann-Whitney test. "x" indicates mean. Lines indicate median and interquartile ranges. ● indicate individual patient values.

**Fig. 2:**
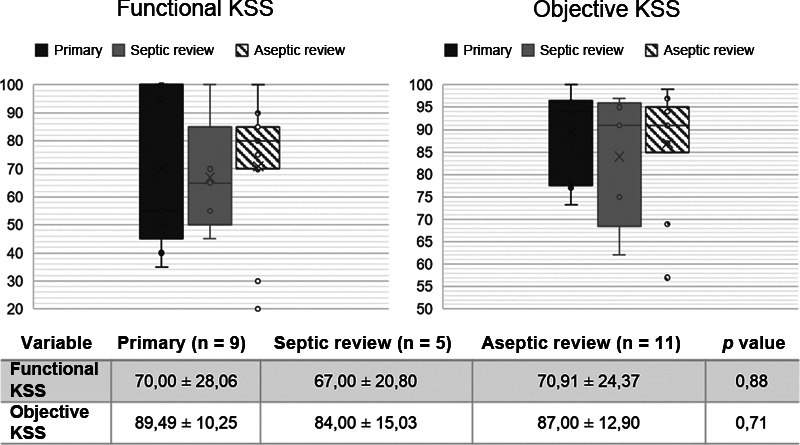
Functional and objective clinical scores (KSS) of patient subgroups who used the constrained Hinge prosthesis. KSS represented in dots, ANOVA test performed. Dark Gray = Primary TKA. Light Gray = Septic Revision. Striped = Aseptic Revision. "x" indicates mean. Lines indicate median and interquartile ranges. ● indicate individual patient values.

The KSS values of the Hinge and control groups are illustrated in Fig. 1 and did not demonstrate a significant difference between the groups. The KSS values for each type of surgery in the Hinge group are shown in Fig. 2 and we did not observe a significant difference between the subgroups.

**Fig. 3:**
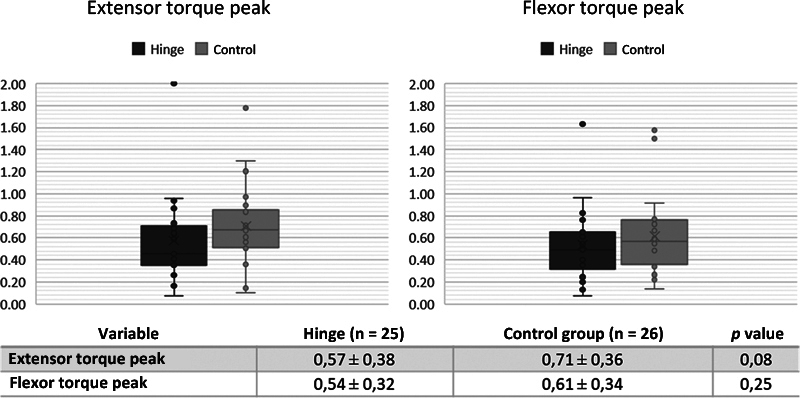
Extensor and flexor torque peak of patients undergoing total knee arthroplasty with Hinge-type prosthesis (Dark Gray) and with prosthesis with posterior stabilization (Control: Light Gray). Peak torque represented in Newton meter / kilo. Mann-Whitney test. "x" indicates mean. Lines indicate median and interquartile ranges. ● indicate individual patient values.

**Fig. 4:**
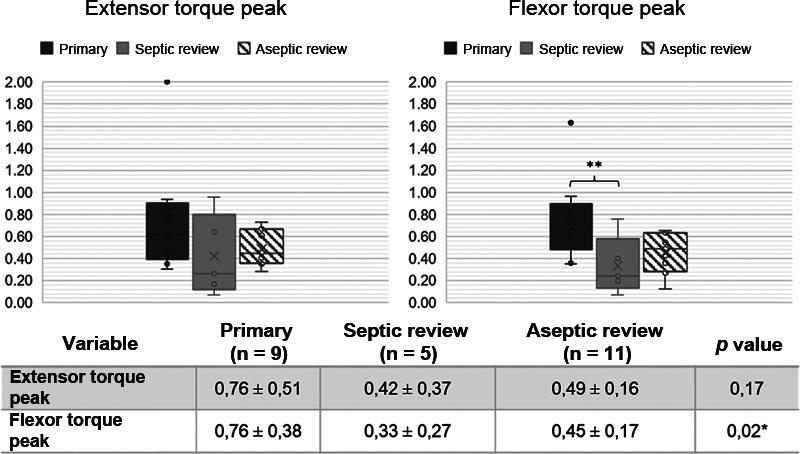
Peak extensor and flexor torque of patient subgroups who used the Hinge-type prosthesis. Peak torque represented in Newton meter / kilo. ANOVA test. * p < 0,05 Dark Gray = Primary TKA. Light Gray = Septic Revision. Striped = Aseptic Revision. "x" indicates mean. Lines indicate median and interquartile ranges. ● indicate individual patient values.

The peak flexor and extensor torque values corrected for weight are illustrated in Fig. 3 and did not demonstrate significant differences between the Hinge group and the control group. Fig. 4 shows the peak flexor and extensor torque between the different types of surgery using Hinge implants. A significant difference was observed between peak flexor torque values (p = 0.02) between the subgroups that used the Hinge implant. After performing the Bonferroni post hoc test, a difference (p < 0.05) was observed between the primary prosthesis and septic revision subgroups; therefore, the group that used the constrained prosthesis in primary arthroplasties has a higher peak flexor torque (0.76 Nm/kg) than those that used the implant in revision TKA after TKA infection (0.33 Nm/kg).

## Discussion

The present study is one of the few found in the literature evaluating results from the use of constrained prostheses in the Brazilian and Latin American populations. 12 13 Contrary to the predicted hypothesis, the main finding was the observation that the use of constrained implants enables joint function and muscle strength comparable to that of patients who underwent primary arthroplasty using conventional implants with posterior stabilization.

In our sample, the average functional KSS was 69.80 points in the Hinge group, not statistically different from the 74.23 points observed in the control group; we also found no differences in values between the subgroups that used constrained implants. This score was higher than that observed by most studies evaluating constrained prostheses found in the literature, which showed results ranging from 36 to 69.7 points. 12 14 15 16 17 18 19 The two trials with values similar to ours were those by Felli et al. 16 (67,1 points) who evaluated revisions and primary arthroplasties in patients with rheumatoid arthritis and Petrou et al. 17 (69,7 points) who evaluated medium-term results of cemented primary constrained prostheses.

Similarly, when analyzing the objective KSS, no differences were observed among the three subgroups of patients who received Hinge prostheses. This demonstrates, in our sample, that the type of surgery, whether primary or revision, did not influence the final outcome of joint function. Similarly, the objective KSS score was not distinct between patients with constrained implants (87.29 points) and those with conventional primary prostheses (89.96 points). Similar data were observed by Sanguineti et al. 18 , Felli et al. 16 , Bohler et al. 19 and Helito et al. 12 , who reported means of 94.2, 93.5, 89.0, and 89.9 points, respectively. Studies conducted by Boelch et al. 14 , Arnholdt et al. 15 and Spranz et al. 20 , obtained lower values, ranging from 67 to 83 points.

Muscular strength and power in the lower limbs are closely associated with physical performance and functional capacity in patients with knee pathologies. Kim et al. 21 demonstrated that the isokinetic peak torque observed preoperatively is recovered one year post-surgery, coinciding with the resumption of full activities by patients. Therefore, we believe that all patients were fully recovered at the time of the evaluation of joint function and limb muscle strength.

We conducted the assessment of muscle strength using isokinetic dynamometry, which allows for a more objective measurement than other methods. It has also been employed in various studies seeking to identify the correlation of muscle strength with different variables, such as surgical access types, anesthesia types, and rehabilitation methods. However, a limited number of studies have specifically aimed to identify the potential influence of the type of joint implant on muscle strength. In our study, we measured strength by peak torque, considering the implant type as the sole variable. We observed that the extensor peak torque was similar between patients in the Hinge group and the control group. Similarly, we did not identify differences among patients with constrained implants, whether in primary or revision surgery. Thus, we did not find that the use of Hinge-type implants influenced the extensor peak torque.

Similarly, the flexion strength measured by peak flexor torque was not different between patients with constricted implants and those with conventional postero-stabilized implants. However, when analyzing only patients in the Hinge group, we identified that patients undergoing primary surgery had higher peak flexor torque than patients undergoing septic revision. We believe that this difference may be related to the potential greater number of surgeries to control septic failure, although we did not identify distinctions regarding peak extensor torque. Pasquier et al. 22 analyzed the use of constrained implants in TKA revision surgeries and demonstrated that the use of the implant in septic revisions presented worse functional results and higher complication rates when compared to cases of aseptic failures.

Recent studies evaluating the long-term durability of the implant demonstrate excellent results, showing a 10-year survival rate for revisions of 90.2% and for primary arthroplasties in patients over 60 years of age of 94%. 8 23 However, as seen in the literature, a relevant percentage of our patients who used Hinge presented poor results and needed to be excluded from the analysis, with three developing arthroplasty failure and two with extensor mechanism failure, totaling 7.46% of the initial sample. Although relevant, this value is lower than the average number of complications observed in arthroplasties using constrained implants, which range from 9.2 to 63%. 10 23 24 25 26 27 28 Most studies demonstrate an incidence of 30 to 40% of complications, the most common being: infections, injuries to the extensor mechanism, and fractures. 10 24 26 29 30

As limitations of our research, we can mention the high number of exclusions, which occurred not only in our study but also in other similar researches that showed a follow-up loss of up to 65%. 18 30 This can be partially explained by the social profile of the patients and the institution where the research was conducted. Many patients operated in our hospital are referred from different regions of the country, making follow-up challenging after several years of surgery. The advanced age and presence of comorbidities in this population often hinder the assessment of muscle strength and contribute to high mortality from various causes in the years following surgery.

Another significant limitation of this study is the lack of control over other variables such as surgical access, although several studies have shown that differences in functional recovery related to surgical access are observed up to 6 months. Kim et al. 21 corroborate this information by demonstrating that the peak muscle torque after one year of surgery is similar to that observed preoperatively.

Finally, we believe that the lack of sample calculation represents an important limitation of the study. However, due to the restricted indications and limited availability of constrained implants, we believe that the analysis of 25 surgeries with constrained rotating platform implants with an isokinetic dynamometer reveals significant information, although it is necessary to carry out future studies with a greater number of cases and complementary analyzes to increase the reliability of results.

## Conclusion

1. The use of constrained implants enables joint function and muscle strength comparable to patients who underwent primary arthroplasty using conventional implants with posterior stabilization.
2. Patients undergoing septic revision with a rotating Hinge prosthesis exhibit lower flexor muscle strength compared to those undergoing primary total knee arthroplasty (TKA) with a constrained implant.
