# Supplementary material for: Comparative Functional and Isokinetic Analysis between Implants with Posterior Stabilization and Rotating Hinge Total Knee Arthroplasty
Source: Rev Bras Ortop (Sao Paulo). 2024 Mar 21;59(1):e68–75. doi: 10.1055/s-0044-1779685 (PMC10957273; doi:10.1055/s-0044-1779685)
Supplement: Related texts

# Research article: Análise funcional e isocinética comparativa entre implantes com estabilização posterior e rotatórios constritos ( *hinge* ) em artroplastias do joelho

**Keywords:** artroplastia do joelho, osteoartrite do joelho, força muscular

## Abstract

**Objetivo:** Comparar a função e a força muscular do membro entre pacientes submetidos a artroplastias do joelho que utilizaram implantes primários com estabilização posterior (grupo controle) e pacientes com implantes constritos rotatórios (grupo Hinge).

**Métodos:** A avaliação da função foi feita por meio do *Knee Society Score* (KSS) e da força muscular por um dinamômetro isocinético utilizando a velocidade de 60°/s.

**Resultados:** Foram analisados 43 pacientes, que realizaram 51 cirurgias, sendo o grupo Hinge composto por 25 cirurgias e o grupo controle por 26 cirurgias primárias. Não observamos diferenças significativas entre os grupos Hinge e controle nos valores do KSS funcional (p = 0,54), KSS objetivo (p = 0,91), pico de torque flexor (p = 0,25) e pico de torque extensor (p = 0,08). Os pacientes do grupo Hinge que realizaram artroplastias primárias apresentaram um pico de torque flexor maior (0,76 Nm/kg) que aqueles que utilizaram o implante em revisão após falha séptica (0,33 Nm/kg) (p < 0,05). O implante constrito foi indicado em cirurgias de revisão de artroplastia com instabilidade ligamentar grave e em casos de artroplastias primárias complexas com destruição óssea ou deformidade coronal grave no plano coronal.

**Conclusão:** O uso de implantes bloqueados possibilita função articular e força muscular comparáveis a dos pacientes que realizaram artroplastia primária utilizando implantes convencionais com estabilização posterior. Pacientes submetidos à revisão séptica com prótese Hinge rotatória apresentam menor força da musculatura flexora em relação àqueles submetidos a artroplastia primária com implante constrito.

## Introdução

A artroplastia total do joelho (ATJ) é um procedimento cirúrgico de substituição articular do joelho por próteses metálicas. É, frequentemente, indicado para o tratamento dos casos graves de osteoartrite, apresentando bons ou excelentes resultados em mais de 90% dos pacientes. Normalmente, para as cirurgias primárias são utilizados implantes de menor constrição, podendo ser retendo ou substituindo o ligamento cruzado posterior. Nos casos de cirurgias primárias complexas ou nos de revisão de ATJ são, mais frequentemente, utilizados implantes com maior grau de constrição, podendo ser semi-constritos ou constritos (implantes tipo Hinge). 1 Atualmente, instabilidade ligamentar grave, destruição óssea maciça, hiperfrouxidão ligamentar, deformidade grave fixa no plano coronal, artrofibrose grave, revisão séptica de artroplastia, artrite reumatoide grave e fratura cominuta em osso osteoporótico podem ser indicação de uso dos implantes tipo Hinge. 2 3

Esses implantes foram desenhados por Walldius, 4 em 1953, para reconstrução após ressecção tumoral. 4 5 Entretanto, as próteses de primeira geração evoluíram com limitado resultado clínico, elevado número de falhas, limitada durabilidade e significativa perda óssea para revisão. 6 Na segunda geração, apesar das melhorias observadas no desenho, o resultado clínico permanecia limitado e, ainda, um alto número de complicações era observado. 7 Atualmente, dispomos da terceira geração desses implantes, com alterações no desenho e utilização de componentes de polietileno rotatório objetivando melhor distribuição de carga e cinemática articular, assim como, redução do estresse na interface implante-osso hospedeiro. 8 Assim sendo, acredita-se que os modernos implantes constritos rotatórios possibilitem melhores resultados clínicos; entretanto, limitado número de estudos avaliam os resultados funcionais desses implantes, sobretudo na população brasileira, assim como, observamos reduzido número de trabalhos comparando os resultados de implantes com diferentes graus de constrição. 9 10

Desse modo, o objetivo do presente estudo foi comparar a função e a força muscular do membro entre pacientes submetidos à cirurgia utilizando implantes primários com estabilização posterior e pacientes com implantes constritos rotatórios. Acreditamos que os pacientes portadores de implantes tipo Hinge apresentarão menor força muscular do membro e/ou pior função articular, potencialmente relacionado ao implante, mas também devido à maior complexidade dos casos primários ou por ser utilizados em pacientes submetidos à revisão de ATJ, portanto, com diversas cirurgias prévias.

## Material e Métodos

De dezembro de 2009 a maio de 2016, 67 implantes constritos rotatórios do sistema *Rotation Hinge Knee (RHK) Zimmer-Biomet* ® foram utilizados em 65 pacientes em nossa instituição. Desses, 18 casos eram artroplastias primárias complexas e 50 eram revisões de ATJ. Para o presente estudo, 24 pacientes, submetidos a 25 cirurgias com implante tipo Hinge, foram analisados, constituindo o “grupo Hinge”. Os critérios de inclusão foram pacientes submetidos a ATJ na instituição que necessitaram de implante constrito tipo Hinge e que possuíam avaliação isocinética. Os critérios de exclusão foram: pacientes com menos de um ano de pós-operatório no período do estudo, pacientes que não cumpriram as orientações de pós-operatório, perda de seguimento, evolução desfavorável com perda do implante (amputação, artrodese), evolução para óbito por qualquer motivo, presença de lesões musculares, lesões neurológicas, lesões do mecanismo extensor e/ou fraturas em membros inferiores, doença clínica sistêmica descompensada e pacientes residentes fora do estado em que foi realizado o estudo. 41 pacientes foram excluídos do trabalho e as causas foram: perda de seguimento (15 pacientes), residência fora do estado (oito pacientes), óbito (seis pacientes), ausência de condições clínicas para avaliação isocinética (cinco pacientes), falha séptica da artroplastia (três pacientes), falta de interesse em participar da pesquisa (dois pacientes) e falha do mecanismo extensor (dois pacientes).

Das 25 cirurgias do grupo Hinge, nove eram artroplastias primárias e 16 revisões de ATJ. Em quatro casos de artroplastias primárias foi necessário o uso de implantes constritos devido à destruição óssea e, em outros cinco casos, devido à deformidade grave no plano coronal. Das 16 revisões, 11 foram devido a falhas mecânicas e cinco devido a infecção.

O grupo controle foi constituído por pacientes submetidos à ATJ com implantes primários convencionais com estabilização posterior com idade, sexo e índice de massa corpórea (IMC) semelhantes ao grupo estudo que utilizou a prótese constrita Hinge.

Portanto, trata-se de uma série de casos retrospectiva realizada com 24 pacientes submetidos a 25 ATJ que utilizaram implantes constritos com plataforma tibial rotatória e por 19 indivíduos que realizaram artroplastias primárias em 26 cirurgias utilizando implantes com estabilização posterior. O estudo foi previamente aprovado pelo Comitê de Ética em Pesquisa da instituição (CAAE: 47473315.4.0000.5273) e não houve limite de idade, nem restrição quanto ao sexo dos participantes.

Em ambos os grupos foram analisados: idade, sexo, tempo de seguimento, peso, altura e IMC. O grupo Hinge foi dividido nos seguintes subgrupos: pacientes submetidos a artroplastia primária (grupo primária), pacientes submetidos a revisão após falha devido a infecção (grupo revisão séptica) e pacientes submetidos a revisão após falha asséptica (grupo revisão asséptica).

As análises descritas a seguir foram realizadas entre o grupo controle e Hinge e entre os subgrupos do grupo Hinge. A avaliação da função foi feita por meio da aplicação do *Knee Society Score* (KSS), validado para a língua portuguesa e que é composto por uma parte objetiva e outra funcional. 11 O KSS objetivo analisa dor, arco de movimento, estabilidade articular, presença de rigidez, déficit de extensão e alinhamento do membro no plano coronal. A parte funcional avalia a mobilidade do paciente, a capacidade de subir e descer escadas e a necessidade do uso de auxílio de instrumentos para deambular. O questionário foi aplicado por dois ortopedistas durante a consulta de acompanhamento pós-operatório.

A força muscular foi analisada através de um dinamômetro isocinético (CSMI, modelo HUMAC NORM). Para determinar a força voluntária isocinética máxima foi realizado teste isocinético concêntrico-concêntrico para flexão e extensão do joelho. A velocidade utilizada foi 60°/s e foram realizadas cinco repetições. O maior torque instantâneo encontrado foi considerado o pico de torque (PT) e utilizado para as análises. O PT foi normatizado pelo peso corporal para possibilitar uma melhor comparação entre os indivíduos.

## Análise estatística

As análises descritivas para os dados quantitativos foram realizadas apresentado médias, desvios padrão (DP), medianas, valores mínimos e máximos. Foi utilizado o teste de normalidade de *Shapiro-Wilk* e teste de homogeneidade de Levene. Para a comparação dos grupos controle e Hinge foi utilizado o teste t para medidas independentes e, quando necessário, o teste não-paramétrico de Mann Whitney. Para analisar as variáveis entre os subgrupos da prótese Hinge foi utilizado o teste de Análise de Variância (ANOVA) e o teste não-paramétrico de Kruskal-Wallis. Quando foi necessário realizar comparações múltiplas de médias, foi utilizado o teste *post hoc* de Bonferroni e o teste *post hoc* de Dunn. As variáveis categóricas foram analisadas com o teste de Qui Quadrado ou Exato de Fisher quando necessário. Todas as análises foram realizadas no software SPSS 21 para Windows com nível de significância de α = 0,05.

## Resultados

Os dados demográficos dos grupos Hinge e controle estão descritos na Tabela 1 e os grupos foram semelhantes quanto à idade, ao peso, à altura, ao IMC, a distribuição do sexo e ao tempo de seguimento.

**Tabela 1:** Dados demográficos e tempo de seguimento dos pacientes submetidos a artroplastia do joelho com implante tipo Hinge e implante com estabilização posterior (grupo controle)

Variável	Hinge (n = 25)	Grupo Controle (n = 26)	p valor
**Idade (anos)**	68,20 ± 9,09	66,40 ± 6,19	0,23
**Sexo**			
** Masculino**	4	5	0,76
** Feminino**	21	21	
**Peso (kg)**	78,42 ± 16,42	78,90 ± 12,95	0,66
**Altura (m)**	1,59 ± 0,10	1,63 ± 0,09	0,27
** IMC (kg/m ^2^ ) **	31,19 ± 6,46	29,51 ± 3,97	0,61
**Tempo de seguimento (meses)**	32,74 ± 21,23	27,91 ± 18,41	0,37

Não houve diferença estatisticamente significativa quanto a distribuição por sexo, a idade e ao tempo de seguimento entre pacientes submetidos à Hinge na cirurgia primária ou na cirurgia de revisão. (A média do IMC apresentou valores diferentes entre os grupos ( *p* = 0,02) ( Tabela 2 ) e, após a realização do teste *pos hoc* de Bonferroni, observou-se p valor < 0,05 somente entre os subgrupos revisão séptica e revisão asséptica; portanto, o subgrupo revisão séptica (38,41 kg/m ^2^ ) apresentou média superior ao subgrupo revisão asséptica (28,37 kg/m ^2^ ).

**Tabela 2:** Dados demográficos e tempo de seguimento dos pacientes submetidos a artroplastia do joelho com implante tipo Hinge e implante com estabilização posterior (Grupo controle)

Variável	Primária (n = 9)	Revisão Séptica (n = 5)	Revisão Asséptica (n = 11)	p valor
**Idade (anos)**	66,73 ± 10,47	71,53 ± 7,02	67,81 ± 9,26	0,23
**Sexo**				
** Masculino**	3	0	1	0,18
** Feminino**	6	5	10	
**Peso (kg)**	75,54 ± 15,46	90,52 ± 22,09	75,27 ± 19,78	0,29
**Altura (m)**	1,57 ± 0,11	1,53 ± 0,10	1,62 ± 0,09	0,26
** IMC (kg/m ^2^ ) **	30,63 ± 6,73	38,41 ± 7,02	28,37 ± 5,43	0,02*
**Tempo de seguimento (meses)**	29,33 ± 25,22	35,51 ± 23,39	34,28 ± 18,33	0,62

Os valores do KSS do grupo Hinge e controle estão ilustrados na Fig. 1 e não demonstraram diferença significativa entre os grupos. Os valores do KSS para cada tipo de cirurgia do grupo Hinge estão apresentados na Fig. 2 e não observamos diferença significativa entre os subgrupos.

**Fig. 1:** Escore clínico funcional e objetivo (KSS) dos pacientes submetidos a artroplastia total do joelho com prótese tipo Hinge (Hinge: cinza escuro) e com prótese com estabilização posterior (Controle: cinza claro) KSS representado em pontos. DP = Desvio padrão. Teste de Mann Whitney “x” indica média. Linhas indicam mediana e intervalos interquartílicos. ● indicam os valores individuais dos pacientes.

**Fig. 2:** Escore clínico funcional e objetivo (KSS) dos subgrupos de paciente que utilizaram prótese constrita Hinge. KSS representado em pontos, realizado teste ANOVA. Cinza Escuro = ATJ Primária. Cinza Claro = Revisão Séptica. Listrado = Revisão Asséptica. “x” indica média. Linhas indicam mediana e intervalos interquartílicos. ● indicam os valores individuais dos pacientes.

Os valores do pico de torque flexor e extensor corrigidos pelo peso estão ilustrados na Fig. 3 e não demonstraram diferenças significativas entre o grupo Hinge e grupo controle. A Fig. 4 apresenta o pico de torque flexor e extensor entre os diferentes tipos de cirurgia utilizando implantes Hinge. Observou-se diferença significativa entre os valores do pico de torque flexor ( *p* = 0,02) entre os subgrupos que utilizaram implante tipo Hinge. Após a realização do teste de *pos hoc* de Bonferroni, observou-se diferença (p < 0,05) entre os subgrupos prótese primária e revisão séptica; portanto, o grupo que utilizou a prótese constrita em artroplastias primárias possui o pico de torque flexor maior (0,76 Nm/kg) que aqueles que utilizaram o implante em revisão de ATJ após infecção da mesma (0,33 Nm/kg).

**Fig. 3:** Pico de torque extensor e flexor dos pacientes submetidos a artroplastia total do joelho com prótese tipo Hinge (Cinza escuro) e com prótese com estabilização posterior (Controle: Cinza claro) Pico de torque representado em Newton metro / quilo. Teste de Mann Whitney “x” indica média. Linhas indicam mediana e intervalos interquartílicos. ● indicam os valores individuais dos pacientes.

**Fig. 4:** Pico de torque extensor e flexor dos dos subgrupos de paciente que utilizaram prótese tipo Hinge Pico de torque representado em Newton metro / quilo. Teste ANOVA. * p < 0,05 Cinza Escuro = ATJ Primária. Cinza Claro = Revisão Séptica. Listrado = Revisão Asséptica. “x” indica média. Linhas indicam mediana e intervalos interquartílicos. ● indicam os valores individuais dos pacientes.

## Discussão

O presente estudo é um dos poucos encontrados na literatura avaliando resultados do uso de prótese constritas na população brasileira e na da América Latina. 12 13 Contrariando a hipótese prevista, o principal achado foi a observação que o uso de implantes constritos possibilita função articular e força muscular comparáveis a dos pacientes que realizaram artroplastia primária utilizando implantes convencionais com estabilização posterior.

Em nossa amostra, a média do KSS funcional foi 69,80 pontos no grupo Hinge, não sendo estatisticamente distintos dos 74,23 pontos observados no grupo controle; também não encontramos diferenças nos valores entre os subgrupos que utilizaram implantes constritos. Essa pontuação foi superior à que foi observada pela maioria dos estudos avaliando próteses constritas encontrados na literatura, que mostraram resultados variando de 36 a 69,7 pontos. 12 14 15 16 17 18 19 Os dois ensaios com valores semelhantes ao nosso foram os de Felli et al. 16 (67,1 pontos) que avaliaram revisões e artroplastias primarias em pacientes com artrite reumatóide e o de Petrou et al. 17 (69,7 pontos) que avaliaram resultados a médio prazo de próteses constritas primárias cimentadas.

De modo similar, ao analisar o KSS objetivo não foram observadas diferenças entre os três subgrupos de pacientes em que foram implantadas próteses Hinge, demonstrando, em nossa amostra, que o tipo de cirurgia, primária ou revisão, não influenciou no resultado final da função articular. Do mesmo modo, a pontuação do KSS objetivo não foi distinta entre os pacientes com implantes constritos (87,29 pontos) e próteses primárias convencionais (89,96 pontos). Dados similares foram observados por Sanguineti et al. 18 , Felli et al. 16 , Bohler et al. 19 e Helito et al. 12 , que encontram médias de 94,2, 93,5, 89,0 e 89,9 pontos respectivamente. Os estudos realizados por Boelch et al. 14 , Arnholdt et al. 15 e Spranz et al. 20 , obtiveram valores menores, variando 67 a 83 pontos.

A força e a potência muscular em membros inferiores estão intimamente associadas a performance física e a capacidade funcional em pacientes com patologias no joelho. Kim et al. 21 demonstraram que o pico de torque isocinético observado no pré- operatório é recuperado após um ano de cirurgia, com a retomada das atividades plenas dos pacientes; assim, acreditamos que todos os pacientes encontravam-se plenamente recuperados no momento da avaliação da função articular e força muscular do membro.

Realizamos a avaliação da força muscular por dinamometria isocinética, o que possibilita a mensuração mais objetiva do que a realizada por outros métodos, sendo, também, empregada em diversos estudos que buscaram identificar a correlação da força muscular com distintas variáveis, tais como, tipos de acesso cirúrgico, tipo de anestesia e métodos de reabilitação. Entretanto, limitados números de estudos planejaram identificar a potencial influência do tipo do implante articular sobre a força muscular. Em nosso estudo, realizamos a mensuração da força por meio do pico de torque considerando o tipo de implante como variável única. Observamos que o pico de torque extensor foi similar entre os pacientes do grupo Hinge e do grupo controle. De modo similar, também não identificamos diferenças entre os pacientes portadores de implantes constritos, seja como cirurgia primária ou de revisão. Assim, não identificamos que o uso de implantes tipo Hinge influenciava o pico de torque extensor.

De modo similar, a força de flexão aferida pelo pico de torque flexor não foi distinta entre os pacientes com implantes constritos e aqueles com implantes convencionais póstero-estabilizados. Entretanto, ao analisar somente os pacientes do grupo Hinge, identificamos que os pacientes submetidos à cirurgia primária apresentaram maior pico de torque flexor que os pacientes submetidos à revisão séptica. Acreditamos que tal diferença possa estar relacionada ao potencial maior número de cirurgias para controle da falha séptica, embora, não tenhamos identificado distinções quanto ao pico de torque extensor. Pasquier et al. 22 analisaram o uso implantes constritos em cirurgias de revisão de ATJ e demonstraram que o uso do implante em revisões sépticas apresentaram piores resultados funcionais e maiores taxas de complicações quando comparados aos casos de falhas assépticas.

Estudos recentes avaliando a durabilidade a longo prazo do implante demonstram ótimos resultados, apresentando sobrevida em 10 anos de revisões de 90,2% e de artroplastias primárias em pacientes com mais de 60 anos de 94%. 8 23 Porém, assim como se verifica na literatura, uma porcentagem relevante dos nossos pacientes que utilizaram Hinge apresentaram maus resultados e necessitaram ser excluídos da análise, sendo que três evoluíram com falha da artroplastia e dois com falha do mecanismo extensor, totalizando 7,46% da amostra inicial. Apesar de relevante, esse valor é menor que a média de complicações observadas em artroplastias utilizando implantes constritos, que variam de 9,2 a 63%. 10 23 24 25 26 27 28 A maioria dos trabalhos demonstram incidência de 30 a 40% de complicações, sendo as mais comuns: infecções, lesões do mecanismo extensor e fraturas. 10 24 26 29 30

Como limitações de nossa pesquisa podemos citar o elevado número de exclusões, que aconteceram não somente do nosso estudo, mas também de outras pesquisas similares que demonstraram perda de seguimento de até 65%. 18 30 Isso pode ser explicado, parcialmente, pelo perfil social dos pacientes e da instituição onde foi realizada a pesquisa. Muitos pacientes operados em nosso hospital são encaminhados de diferentes regiões do país, o que dificulta o seguimento após alguns anos de cirurgia. A idade elevada e presença de comorbidades dessa população impossibilitam, em muitos casos, a avaliação da força muscular; assim como, culminam com elevada mortalidade por diversas causas nos anos subsequentes à cirurgia.

Outra importante limitação desse estudo refere-se ao não controle de outras variáveis como o acesso cirúrgico, embora, diversos trabalhos tenham demonstrados que diferenças na recuperação funcional relativas ao acesso cirúrgico são observadas até 6 meses. Kim et al. 21 corroboram essa informação demonstrando que o pico de torque muscular após um ano de cirurgia é similar ao observado no pré-operatório.

Por fim, acreditamos que a ausência de cálculo amostral representa importante limitação do estudo. Contudo, devido às indicações restritas e limitada disponibilidade dos implantes constritos, acreditamos que a análise de 25 cirurgias com implantes constritos de plataforma rotatória com dinamômetro isocinético revelem significativas informações, embora seja necessário a realização de estudos futuros com maior número de casos e análises complementares para aumentar a confiabilidade dos resultados.

## Conclusões

1. O uso de implantes bloqueados possibilita função articular e força muscular comparáveis a dos pacientes que realizaram artroplastia primária utilizando implantes convencionais com estabilização posterior.
2. Pacientes submetidos à revisão séptica com prótese Hinge rotatória apresentam menor força da musculatura flexora em relação àqueles submetidos a ATJ primária com implante bloqueado.
